# What is the cost of dedication to teaching? A school-based longitudinal quantitative study on habits and quality of life

**DOI:** 10.1371/journal.pone.0359001

**Published:** 2026-09-29

**Authors:** Isabela Fernanda Rodrigues de Oliveira, Suelen Marçal Nogueira, Flávio Manoel Coelho Borges Cardoso, Gabriel Barg, Gustavo De Conti Teixeira Costa, Emilio Villa-González, Priscilla Rayanne e Silva, Matias Noll

**Affiliations:** 1 Department of Public Health, Instituto Federal Goiano, Goiás, Brazil; 2 Department of Health Sciences, Universidade Federal de Jataí, Jataí, Goiás, Brazil; 3 Department of Neuroscience and Learning, Universidad Católica del Uruguay, Montevideo, Uruguay; 4 Departments of Nutrition and Health, and of Physical Education, Universidade Federal de Goiás, Goiania, Goiás, Brazil; 5 Department of Physical Education and Sports, Faculty of Sport Sciences, Sport and Health University Research Institute (iMUDS), University of Granada, Granada, Spain; 6 Institute of Cognitive and Clinical Neurosciense, Central Institute of Mental Health, Medical Faculty Mannheim, Heidelberg University, Mannheim, Germany; Chulabhorn Royal Academy, THAILAND

## Abstract

This study aims to evaluate, through a longitudinal research, the association between lifestyle habits, modifiable risk behaviors, and mental health with quality of life (QoL) among professionals of the Federal Network of Professional, Scientific, and Technological Education (education professionals) in Brazil. A total of 600 professionals (including teachers and administrative technicians) were longitudinally evaluated in 2022 and 2023. Four validated questionnaires were used: the WHOQOL-bref to assess quality of life (QoL), the DASS-21 for mental health, the Back-PEI for physical activity, and PeNSE for dietary markers. The statistical analysis included Relative Risk (*RR*) tests and adjusted analysis using a Poisson regression model with robust variance. The main findings indicate that women exhibit poorer QoL in the physical domain (*RRadj* = 1.19, p = 0.005) and psychological domain (*RRadj* = 1.14, p = 0.045). Being employed and over 60 years old is a protective factor for QoL in the psychological domain (*RRadj* = 0.67; p < 0.05). The regular consumption of ultraprocessed foods is a risk factor for QoL all domains (*RRadj* > 1.15, p < 0.05). Irregular consumption of vegetables/greens (*RRadj* > 1.11, p < 0.05) and fruits (*RRadj* > 1.10, p < 0.05) indicated a risk of low QoL. Participants with low frequency or no physical activity had a higher risk of worse quality of life scores across all domains (*RRadj* > 1.20; p < 0.001). Exposure to television for 5 hours or more during the day was a risk factor for low QoL in the physical domain (*RRadj* = 1.33, p = 0.005) and social relationships (*RRadj* = 1.32, p = 0.003). Computer use was not associated with QoL (p > 0.05). Higher frequency of alcohol consumption was associated with higher scores in the social relationships domain (*RRadj* = 0.83, p = 0.037). Regarding mental health, depressive, anxiety, and stress symptoms were associated with low QoL across all domains (*RRadj* > 1.33, p < 0.01) in both men and women. The results of this longitudinal study may encourage healthier lifestyle habits and support public policies aimed at improving the well-being of education professionals.

## Introduction

Quality of life (QoL) represents a constant challenge in public health, as it is necessary to understand its impacts across different dimensions of everyday life: physical, psychological, social, and environmental [1]. QoL not only determines the health status but also autonomy, satisfaction, longevity, and the individual’s ability to adapt to changes and life’s challenges [2]. Mental disorders reduce QoL, leading to functional impairment, social exclusion, and an increased risk of concomitant physical illnesses [3]. The impact of mental health is particularly high for depressive and anxiety disorders, which are the leading causes of functional disability [4]. QoL is shaped by a wide set of interrelated factors, including living conditions (housing quality, neighborhood and environmental safety), access to services (health, education, transportation, and public services), education, and leisure opportunities [5,6]. Furthermore, safe, welcoming, and balanced work environments significantly contribute to the improvement of well-being and QoL [7].

In the context of professionals, QoL is determined by a combination of collaborative environments, fair rewards, supportive relationships, stability, and a balance between personal and professional life [8]. Organizational culture and interpersonal dynamics constitute pivotal determinants in the establishment of psychologically safe and inclusive physical and social environments [9]. Supportive leadership, communication, team spirit, and opportunities for participation in decision-making further promote satisfaction in the work environment and a sense of belonging; therefore, these factors are considered determinants of QoL [10]. Work environments with high demands, such as those for education professionals, contribute to an increase in symptoms of anxiety, depression, and burnout [11]. Precarious working conditions, such as excessive workload, lack of available resources, low salaries, and insufficient recognition, are factors that wear down the mental health and well-being of educational professionals [12].

Lifestyle habits have gained prominence in the literature as one of the determinants of Quality of Life (QoL) in all its dimensions [13,14]. Habits related to nutrition, physical activity, screen time, and modifiable risk behaviors, such as smoking and alcohol consumption, are examples of lifestyle habit variables [15,16]. Some studies show that unhealthy eating patterns, especially high consumption of ultra-processed foods (UPF), such as soft drinks, packaged snacks, processed meats, and ready-to-eat meals, are directly linked to low QoL [17,18]. Regular consumption of these foods can increase fatigue, symptoms of anxiety and depression, and also pose a higher risk of chronic diseases such as obesity, diabetes, and cardiovascular diseases [19,20]. Conversely, the practice of regular physical activity is consistently associated with better physical and mental QoL, greater vitality, and a lower risk of disability and mortality [21,22]. Due to the exhausting routines of education professionals, time and energy are limited for practicing healthy habits, such as maintaining a balanced diet with regular meal times, engaging in physical activity frequently, including the maintenance of optimal sleep hygiene and sufficient sleep duration [23,24]. Screen exposure increased significantly due to the COVID-19 pandemic, especially among this group of education professionals, who had to replace in-person classes with online modalities [25].

Few longitudinal studies have been conducted in Latin America addressing the factors associated with quality of life (QoL) among education professionals. In this regard, in Brazil, we have the Federal Network of Professional, Scientific and Technological Education (education professionals), which aims to train students at various levels, offering technical-professional education, undergraduate and postgraduate programs (specialization/MBA, master’s, doctorate, postdoctoral). The goal of the education professionals is to promote public, free, and high-quality education throughout the Brazilian territory, integrating teaching, research, and extension activities [26]. Occupational, psychosocial, and lifestyle factors have been associated with the Quality of Life (QoL) of education professionals through national and international research [27,28]. However, these studies have a cross-sectional design [27,28], limiting the understanding of the progression of these factors over time, especially in the context of education professionals. Thus, investigating these determinant factors prospectively may contribute to the development of institutional strategies and public policies aimed at education professionals.

Based on what has been presented, this research aims to contribute to the understanding of quality of life among education professionals, filling the gap regarding quality of life through a longitudinal follow-up. Starting from this assumption, the objective of the present study is to evaluate, through a longitudinal study, the association between lifestyle variables, modifiable risk behaviors, mental health, and quality of life (QoL) among education professionals. The importance of this research lies in the possibility that the results will enable an understanding of the actual demands of professionals in the federal public education sector in Brazil, while also offering insights that may be applicable to educational professionals in other national contexts.

## Method

### Study design and contextualization

This is a longitudinal study with a quantitative approach, conducted at two different time points with the same sample. The first data collection took place from June to November 2022, and the second from September to December 2023, involving from education professionals. The approximately one-year follow-up period was chosen as a relevant interval for detecting potential changes in quality of life, particularly in the context of the post-COVID-19 transition, which involved the resumption and reorganization of in-person educational activities and work routines. This interval also provided a feasible timeframe for participant follow-up. This study is part of a larger project entitled " Quality of Life in Education in Brazil - QoLE-BRA " [29,30]. Due to the involvement of human participants, this research project was approved by the Research Ethics Committee of the Federal Institute of Goiás (Protocol CAAE No. 52353621.3.0000.0036). All participants provided their consent to participate in the study by signing the Free and Informed Consent Form (TCLE).

### Population and sample

The Federal Network was created based on Law No. 11,892 of December 29, 2008, and is managed by the Ministry of Education. In Brazil, the Federal Network were created to expand access to professional and technical education, aiming to democratize knowledge and promote innovation and research. A variety of free courses are offered, including qualification courses, technical programs, undergraduate, and postgraduate studies. The education professionals include 38 Federal Institutes (IFs), 2 CEFETs, 22 technical schools linked to universities, 1 Technological University (UTFPR), and the Pedro II College, totaling 64 educational institutions [31].

The study population consists of 81,812 education professionals from this Federal Network, according to the Nilo Peçanha platform of 2021 [32]. The study included teaching staff and administrative technical professionals (public employees) who were actively working at education professionals, from the five regions of Brazil: Central-West, Northeast, North, Southeast, and South. The exclusion criterion was incomplete questionnaire responses and/or disagreement with the terms of the research. The inclusion criteria comprised education professionals actively working in network institutions, whose email addresses were available on institutional websites; professionals on medical leave or any other form of institutional leave were not included.

The sampling frame consisted of institutional e-mail addresses of education professionals identified through publicly available information on the websites of institutions within the Federal Network. A non-probability sampling approach based on voluntary participation was used. Invitations were distributed to eligible professionals whose institutional e-mail addresses could be identified through these sources. Recruitment was conducted exclusively online through an invitation e-mail containing information about the study and a link to the questionnaire, followed by reminder e-mails throughout the data collection period. To determine the required sample size, a confidence level of 95%, a margin of error of 4%, and an expected proportion of 0.5 were adopted, resulting in a minimum number of 596 respondents, a size considered sufficient to ensure statistical representativeness and allow for the generalization of findings [33].

During the initial data collection (baseline, 2022), a total of 1,566 individuals were recruited. After excluding three cases due to the absence of signed Informed Consent Forms (ICF), the baseline sample comprised 1,563 participants. This figure substantially exceeded the minimum required sample size, ensuring a robust initial dataset. In 2023, we conducted the survey again, and in this stage, the sample consisted of 600 education professionals. Consequently, for the longitudinal analysis, a final subsample of 600 professionals was established, consisting exclusively of those who participated in both waves of data collection.

### Data collection procedure

The questionnaire was administered via Google Forms and sent to the emails of the teachers and administrative technicians. The participants completed an electronic questionnaire containing semi-structured questions. The questionnaire was divided into five sections, with four containing validated questionnaires: (1) sociodemographic questions developed by the authors, (2) World Health Organization Quality of Life Brief Version (WHOQOL-bref), (3) National School Health Survey (PeNSE), (4) Back Pain and Body Posture Evaluation Instrument (Back-PEI), and (5) Depression, Anxiety, and Stress Scale (DASS-21).

The sociodemographic questionnaire included questions about sex (female and male), age (categorized into age groups ≤39, 40–59, and ≥60 years), marital status (single, married, divorced, or widowed), education level (high school, undergraduate, technical-professional education (ETP), Specialization/MBA, Master’s, Doctorate, and Postdoctoral), region (the North and Northeast regions were combined to increase statistical power), and position (faculty or administrative technician).

The validated WHOQOL-bref questionnaire is an abbreviated version consisting of 26 questions related to the physical domain (7 items), psychological (6 items), social relationships (3 items), and environment (8 items) to assess quality of life (QoL), with robust international validation and broad applicability across different contexts and populations [34,35]. For this, a 5-point Likert scale is used, where higher scores correspond to better QoL. In the field testing and validation study of the Portuguese version of the WHOQOL-BREF in Brazil, Fleck et al. (2000) reported satisfactory internal consistency, with a Cronbach’s alpha coefficient of 0.91 for the complete 26-item instrument, and coefficients of 0.84 for the physical domain, 0.79 for the psychological domain, 0.69 for the social relationships domain, and 0.71 for the environment domain [35].

Each domain represents a different aspect of life. The physical domain assesses physical functioning, energy, pain, sleep, mobility, and dependence on medications. The psychological domain encompasses positive and negative feelings, self-esteem, thinking, learning, memory, concentration, body image, and spirituality. The social relationships domain measures satisfaction with personal relationships, social support, and sexual life. Finally, the environment domain assesses aspects of the physical environment, safety, financial resources, access to services, leisure, transportation, and the domestic environment [34,36–39]. The WHOQOL-BREF uses a transformed scale from 0 to 100, where higher scores indicate a better perception of quality of life, reflecting greater satisfaction and well-being of the participants.

The National School Health Survey (PeNSE) is a health surveillance instrument [40], used across various age groups [41,42]. In this research, we used only questions from this questionnaire that relate to diet over the past 7 days and modifiable risk behaviors over the past 30 days. We consider these questions as indicators of lifestyle habits. In the dietary questions, we included the consumption of ultra-processed foods, soft drinks, sweets, fried salty snacks, fast food, legumes, vegetables, and fruits. These variables were categorized into consumption of 0–4 days (not regular) and 5–7 days (regular). In questions about modifiable behaviors, we included items on cigarette consumption (yes/no), alcohol intake (no days, 1–2 days, 3–9 days, 10 days or more), and the number of glasses consumed on the day they drank (none, less than 1 glass/dose, 1–2 glasses/doses, 3–4 glasses/doses, 5 or more glasses/doses).

The Back Pain and Body Posture Evaluation Instrument (Back-PEI) questionnaire was developed to assess the prevalence of back pain and postural, behavioral, and socioeconomic risk factors. It was initially designed for children and adolescents and later adapted for adults [43,44]. The questionnaire has demonstrated content validity based on expert assessment (>90% agreement) and high test-retest reliability among Brazilian schoolchildren, with Cohen’s kappa coefficients ranging from “good” (0.60–0.80) to “very good” (>0.80) for behavioral and postural variables. We used from this questionnaire the questions about physical activity (yes/no), weekly frequency of physical activity (none, 1–2 days, 3–4 days, 5 or more days), time watching television (2 hours or less, 3–4 hours, 5 hours or more), and time using a computer (2 hours or less, 3–4 hours, 5 hours or more).

Finally, for mental health assessment, the DASS-21 was used. It consists of 21 questions and was developed to screen for symptoms of depression, anxiety, and stress across various cultural and population contexts. In the adaptation and validation study of the DASS-21 for Brazilian Portuguese, Vignola and Tucci (2014) reported good internal consistency, with Cronbach’s alpha coefficients of 0.92 for depression, 0.86 for anxiety, and 0.90 for stress [45]. Systematic reviews and multicenter studies demonstrate that the DASS-21 exhibits robust validity and reliability across different countries, languages, and contexts, including Brazil, Malaysia, South Africa, Nigeria, Indonesia, Ghana, the United Kingdom, Finland, and others [45–48]. Each subscale (depression, anxiety, stress) contains seven items and uses a 4-point scale: (0) applied to a small extent or for a short period, (1) applied to a considerable degree or for a significant part of the time, (2) applied very much or most of the time, and (3) not applied at all [45]. To calculate the final scores, it is initially necessary to sum the items of each subscale and multiply by 2 to correspond to the original scale score of the DASS-42. For depression, the scores defined by author Lovibond [49], are: 0–9 (normal), 10–13 (mild), 14–20 (moderate), 21–27 (severe), and 28 or more (extremely severe). For anxiety, the final scores are: 0–7 (normal), 8–9 (mild), 10–14 (moderate), 15–19 (severe), and 20 or more (extremely severe). For stress, the scores are: 0–14 (normal), 15–18 (mild), 19–25 (moderate), 26–33 (severe), and 34 or more (extremely severe).

### Statistical analysis

The normality of the data was assessed using the Shapiro-Wilk test. The paired t-test was performed to compare the means by biological sex between the years 2022 and 2023. The outcome variables (physical domain, psychological, social relationships, environment, and total score) were dichotomized into low QoL and high QoL, defined as values below and above the median, respectively. It is important to note that the median was calculated separately for each domain and for each study wave.

The Relative Risk (*RR*) was calculated to assess the association between sociodemographic variables, lifestyle habits, modifiable risk behaviors, mental health, and QoL stratified by domains. The analyses were conducted in two steps. First, bivariate analyses were performed to estimate unadjusted associations between each independent variable and the outcomes. Variables with a p-value < 0.20 in the bivariate analyses were subsequently included in the adjusted regression models. Sociodemographic questions such as sex, age group, education level, region, and position were used as adjustment variables. Both analyses were conducted using a Poisson regression model with robust variance through Generalized Estimating Equations (GEE), a statistical technique for analyzing correlated data such as repeated measurements from the same individual over time, commonly used in longitudinal studies. GEEs have an exchangeable correlation structure to account for the binary nature of our data [50]. For the evaluation of the statistical significance of the parameters, the Wald test was used, and a significance level of 5% (α = 0.05) was adopted for all tests. The analyses were performed using the *Jamovi* software.

## Results

The sociodemographic data showed that the education professionals analyzed were predominantly female (58.3%), aged between 40 and 59 years (56.7%), single (62.8%), and teachers (59.8%). There was an increase of 1.5% in the number of professionals with a Doctorate or Post-Doctorate degree in the second data collection compared to the baseline, and 3.3% of them managed to advance their educational level to a Master’s degree. Regionally, during the interval between the data collections, only small percentage variations were observed. Among dietary habits, it was observed that regular consumption of ultra-processed foods (UPF) was reported by 14.3% of the professionals in 2022 and 12.7% in 2023. The majority of professionals reported regular consumption of legumes and vegetables, with 70.5% in 2022 and 69% in 2023. It was observed a 7.2% increase in the practice of physical activity between the two data collection periods. Regarding screen time, two opposing trends can be observed: while most professionals watch television for less than 1 hour per day (31.2% in 2022 and 38% in 2023), a smaller proportion watches 5 hours or more, a pattern that will be further explored in the regression analysis. Additionally, the majority also spend 5 hours or more per day using the computer (79% in 2022 and 76.7% in 2023) (Table 1).

**Table 1:** Descriptive distribution of low QoL by WHOQOL-bref domains education professionals, according to the two waves of data collection (2022–2023), Brazil (N = 600).

Variables	Total n (%)		Physical Domain – Low QoL – n (%)		Psychological Domain – Low QoL – n (%)		Social Relations Domain – Low QoL – n (%)		Environment Domain – Low QoL – n (%)		Total Score – Low QoL – n (%)	
Sociodemographic	**2022**	**2023**	**2022**	**2023**	**2022**	**2023**	**2022**	**2023**	**2022**	**2023**	**2022**	**2023**
**Sex**												
Male	350 (58.3)	350 (58.3)	109 (43.6)	137 (54.8)	134 (53.6)	118 (47.2)	165 (66.0)	142(56.8)	133 (53.2)	135 (54.0)	109 (43.6)	125 (50.0)
Female	250 (41.7)	250 (41.7)	209 (59.7)	202 (57.7)	216 (61.7)	196 (56.0)	231 (66.0)	194 (55.4)	189 (54.0)	204 (58.3)	209 (59.7)	179 (51.1)
**Age group**												
≤39	261 (43.5)	226 (37.7)	137 (52.5)	133 (58.8)	167 (64.0)	126 (55.8)	169 (64.8)	132 (58.4)	140 (53.6)	134 (59.3)	137 (52.5)	128 (56.6)
40–59	311 (51.8)	340 (56.7)	170 (54.7)	193 (56.8)	173 (55.6)	176 (51.8)	211 (67.8)	186 (54.7)	174 (55.9)	191 (56.2)	170 (54.7)	163 (47.9)
≥60	28 (4.7)	34 (5.7)	11 (39.3)	13 (38.2)	10 (35.7)	12 (35.3)	16 (57.1)	18 (52.9)	8 (28.6)	14 (41.2)	11 (39.3)	13 (38.2)
**Material status**												
Single	378 (63.0)	377 (62.8)	79 (51.0)	86 (56.2)	85 (54.8)	86 (56.2)	244 (64.6)	218 (57.8)	198 (52.4)	214 (56.8)	79 (51.0)	81 (52.9)
Married	67 (11.2)	70 (11.6)	198 (52.4)	217 (57.6)	224 (59.3)	190 (50.4)	107 (69.0)	85 (55.6)	84 (54.2)	85 (55.6)	198 (52.4)	194 (51.5)
Divorced/Widowed	155 (25.8)	153 (25.5)	41 (61.2)	36 (51.4)	41 (61.2)	38 (54.3)	45 (67.2)	33 (47.1)	40 (59.7)	40 (57.1)	41 (61.2)	29 (41.4)
**Educational level**												
Doctarate/Postdoctoral	133 (22.2)	142 (23.7)	71 (53.4)	83 (58.5)	66 (49.6)	63 (44.4)	82 (61.7)	77 (54.2)	60 (45.1)	71 (50.0)	71 (53.4)	60 (42.3)
Master’s degree	240 (40.0)	260 (43.3)	121 (50.4)	137 (52.7)	145 (60.4)	126 (48.5)	148 (61.7)	140 (53.8)	118 (49.2)	138 (53.1)	121 (50.4)	129 (49.6)
Lato Sensu/MBA	184 (30.7)	164 (27.3)	108 (58.7)	100 (61.0)	115 (62.5)	103 (62.8)	136 (73.9)	100 (61.0)	118 (49.2)	110 (67.1)	108 (58.7)	95 (57.9)
HS/HE/TPE*	43 (7.2)	34 (5.7)	18 (41.9)	19 (55.9)	24 (55.8)	22 (64.7)	30 (69.8)	19 (55.9)	322 (53.7)	20 (58.8)	18 (41.9)	20 (58.8)
**Region**												
South	108 (18.0)	94 (15.7)	48 (44.4)	57 (60.6)	60 (55.6)	43 (45.7)	73 (67.6)	47 (50.0)	55 (50.9)	47 (50.0)	52 (48.1)	44 (46.8)
Southeast	169 (28.2)	166 (27.7)	93 (55.0)	90 (54.2)	100 (59.2)	91 (54.8)	112 (66.3)	95 (57.2)	90 (53.3)	91 (54.8)	86 (50.9)	82 (49.4)
North	24 (4.0)	35 (5.8)	15 (62.5)	24 (68.6)	14 (58.3)	23 (65.7)	14 (58.3)	23 (65.7)	16 (66.7)	24 (68.6)	13 (54.2)	23 (65.7)
Northeast	122 (20.3)	137 (22.8)	72 (59.0)	84 (61.3)	82 (67.2)	76 (55.5)	89 (73.0)	84 (61.3)	73 (59.8)	91 (66.4)	63 (51.6)	79 (57.7)
Midwest	177 (29.5)	168 (28.0)	90 (50.8)	84 (50.0)	94 (53.1)	81 (48.2)	108 (61.0)	87 (51.8)	88 (49.7)	86 (51.2)	86 (48.6)	76 (45.2)
**Position**												
Teacher	359 (59.8)	359 (59.8)	126 (52.3)	135 (56.0)	128 (53.1)	106 (44.0)	149 (61.8)	128 (53.1)	105 (43.6)	117 (48.5)	126 (52.3)	106 (44.0)
Administrative-technical staff	241 (40.2)	241 (40.2)	192 (53.5)	204 (56.8)	222 (61.8)	208 (57.9)	247 (68.8)	208 (57.9)	217 (60.4)	222 (61.8)	192 (53.5)	198 (55.2)
Lifestyle Habits												
Unhealthy dietary intake – considering the past 7 days												
**UPF**												
0 days	119 (19.8)	123 (20.5)	59 (49.6)	67 (60.0)	64 (53.8)	56 (45.5)	79 (66.4)	66 (53.7)	56 (47.1)	70 (56.9)	59 (49.6)	55 (44.7)
1 day	130 (21.7)	166 (27.7)	57 (43.8)	80 (48.2)	66 (50.8)	88 (53.0)	78 (60.0)	77 (46.4)	69 (53.1)	82 (49.4)	57 (43.8)	70 (42.2)
2 days	120 (20.0)	115 (19.2)	62 (51.7)	64 (55.7)	71 (59.2)	53 (46.1)	78 (65.0)	69 (60.0)	61 (50.8)	60 (52.2)	57 (47.5)	58 (50.4)
3 days	91 (15.2)	80 (13.3)	55 (60.4)	45 (56.3)	56 (64.5)	48 (60.0)	63 (69.2)	50 (62.5)	50 (54.9)	47 (58.8)	46 (50.5)	45 (56.3)
4 days	54 (9.0)	40 (6.7)	27 (50.0)	30 (75.0)	29 (53.7)	24 (60.0)	38 (70.4)	27 67.5)	32 (59.3)	27 (67.5)	28 (51.9)	28 (70.0)
5 days	32 (5.3)	34 (5.7)	23 (71.9)	21 (61.8)	25 (78.1)	20 (58.8)	23 (71.9)	23 (67.6)	20 (62.5)	21 (61.8)	20 (62.5)	21 (61.8)
6 days	18 (3.0)	15 (2.5)	10 (55.6)	9 (60.0)	13 (72.2)	6 (40.0)	13 (72.2)	6 (40.0)	9 (50.0)	10 (66.7)	10 (55.6)	8 (53.3)
7 days	36 (6.0)	27 (4.5)	25 (69.4)	23 (85.2)	26 (72.2)	19 (70.4)	24 (66.7)	18 (66.7)	25 (69.4)	22 (81.5)	23 (63.9)	19 (70.4)
**Soft drinks**												
0 days	249 (41.5)	259 (43.2)	129 (51.8)	137 (52.9)	147 (59.0)	138 (53.3)	165 (66.3)	145 (56.0)	134 (53.8)	144 (55.6)	123 (49.4)	129 (49.8)
1 day	131 (21.8)	116 (19.3)	62 (47.3)	66 (56.9)	72 (55.0)	51 (44.0)	82 (62.6)	67 (57.8)	65 (49.6)	70 (60.3)	63 (48.1)	56 (48.3)
2 days	90 (15.0)	105 (17.5)	47 (52.2)	63 (60.0)	52 (57.8)	54 (51.4)	61 (67.8)	50 (47.6)	49 (54.4)	52 (49.5)	42 (46.7)	47 (44.8)
3 days	62 (10.3)	49 (8.2)	38 (61.3)	29 (59.2)	37 (59.7)	25 (51.0)	44 (71.0)	26 (53.1)	36 (58.1)	27 (55.1)	37 (59.7)	25 (51.0)
4 days	22 (3.7)	25 (4.2)	12 (54.5)	19 (76.0)	16 (72.7)	19 (76.0)	15 (68.2)	19 (76.0)	17 (77.3)	20 (80.0)	15 (68.2)	21 (84.0)
5 days	19 (3.2)	15 (2.5)	11 (57.9)	9 (60.0)	12 (63.2)	10 (66.7)	13 (68.4)	8 (53.3)	8 (42.1)	7 (46.7)	8 (42.1)	9 (60.0)
6 days	4 (0.7)	10 (1.7)	2 (50.0)	5 (50.0)	3 (75.0)	6 (60.0)	2 (50.0)	7 (70.0)	1 (25.0)	6 (60.0)	2 (50.0)	6 (60.0)
7 days	23 (3.8)	21 (3.5)	17 (73.9)	11 (52.4)	11 (47.8)	11 (52.4)	14 (60.9)	14 (66.7)	12 (52.2)	13 (61.9)	10 (43.5)	11 (52.4)
**Sweets**												
0 days	78 (13.0)	34 (10.0)	37 (47.4)	34 (52.3)	37 (47.4)	27 (41.5)	48 (61.5)	39 (60.0)	40 (51.3)	32 (49.2)	37 (47.4)	28 (43.1)
1 day	102 (17.0)	107 (17.8)	48 (47.1)	56 (52.3)	59 (57.8)	46 (43.0)	66 (64.7)	55 (51.4)	54 (52.9)	62 (57.9)	47 (46.1)	53 (49.5)
2 days	102 (17.0)	116 (19.3)	45 (44.1)	57 (49.1)	49 (48.0)	56 (48.3)	57 (55.9)	56 (48.3)	47 (46.1)	59 (50.9)	47 (46.1)	52 (44.8)
3 days	102 (17.0)	112 (18.7)	63 (61.8)	67 (59.8)	69 (67.6)	69 (61.6)	74 (72.5)	68 (60.7)	66 (64.7)	62 (55.4)	56 (54.9)	61 (54.5)
4 days	56 (9.3)	57 (9.5)	34 (60.7)	40 (70.2)	33 (58.9)	35 (61.4)	41 (73.2)	34 (59.6)	33 (58.9)	36 (63.2)	30 (53.6)	32 (56.1)
5 days	49 (8.2)	40 (6.7)	28 (57.1)	18 (45.0)	29 (59.2)	22 (55.0)	32 (65.3)	26 (65.0)	20 (40.8)	26 (65.0)	26 (53.1)	22 (55.0)
6 days	28 (4.7)	25 (4.2)	19 (67.9)	19 (76.0)	21 (75.0)	14 (56.0)	22 (78.6)	16 (64.0)	13 (46.4)	15 (60.0)	16 (57.1)	16 (64.0)
7 days	83 (13.8)	78 (13.0)	44 (13.8)	48 (64.5)	53 (63.9)	45 (57.7)	56 (67.5)	42 (53.8)	49 (59.0)	47 (60.3)	41 (49.4)	40 (51.3)
**Fried snacks**												
0 days	189 (31.5)	222 (37.0)	87 (46.0)	123 (55.4)	95 (50.3)	108 (48.6)	120 (63.5)	112 (50.5)	91 (48.1)	106 (47.7)	84 (44.4)	94 (42.3)
1 day	211 (35.2)	218 (36.3)	109 (51.7)	114 (52.3)	126 (59.7)	114 (52.3)	136 (64.5)	123 (56.4)	109 (51.7)	133 (61.0)	102 (48.3)	117 (53.7)
2 days	109 (18.2)	87 (14.5)	62 (56.9)	54 (62.1)	67 (61.5)	48 (55.2)	76 (69.7)	51 (58.6)	59 (54.1)	48 (55.2)	55 (50.5)	44 (50.6)
3 days	50 (8.3)	50 (8.3)	34 (68.0)	31 (62.0)	35 (70.0)	28 (56.0)	36 (72.0)	33 (66.0)	38 (76.0)	34 (68.0)	34 (68.0)	30 (60.0)
4 days	23 (3.8)	13 (2.2)	15 (65.2)	10 (76.9)	16 (69.6)	9 69.2)	17 (73.9)	11 (84.6)	14 (60.9)	10 (76.9)	16 (69.6)	11 (84.6)
5 days	11 (1.8)	6 (1.0)	6 (54.5)	5 (83.3)	6 (54.5)	3 (50.0)	7 (63.6)	3 (50.0)	7 (63.6)	5 (83.3)	6 (54.5)	4 (66.7)
6 days	4 (0.7)	3 (0.5)	3 (75.0)	1 (33.3)	3 (75.0)	3 (100.0)	3 (75.0)	2 (66.7)	2 (50.0)	2 (66.7)	2 (50.0)	3 (100.0)
7 days	3 (0.5)	1 (0.2)	2 (66.7)	1 (100.0)	2 (66.7)	1 (100.0)	1 (33.3)	1 (100.0)	2 (66.7)	1 (100.0)	1 (33.3)	1 (100.0)
***Fast food*:**												
0 days	260 (43.3)	275 (45.8)	129 (49.6)	155 (56.4)	140 (53.8)	135 (49.1)	173 (66.5)	145 (52.7)	140 (53.8)	143 (52.0)	129 (49.6)	126 (45.8)
1 day	185 (30.8)	189 (31.5)	93 (50.3)	102 (54.0)	106 (57.3)	95 (50.3)	115 (62.2)	110 (58.2)	92 (49.7)	106 (56.1)	82 (44.3)	98 (51.9)
2 days	95 (15.8)	76 (12.7)	58 (61.1)	46 (60.5)	65 (68.4)	48 (63.2)	68 (71.6)	43 (56.6)	53 (55.8)	45 (59.2)	52 (54.7)	39 (51.3)
3 days	32 (5.3)	32 (5.3)	22 (68.8)	19 (59.4)	24 (75.0)	20 (62.5)	24 (75.0)	22 (68.8)	22 (68.8)	26 (81.3)	23 (71.9)	22 (68.8)
4 days	15 (2.5)	12 (2.0)	8 (53.3)	5 (41.7)	8 (53.3)	7 (58.3)	10 (66.7)	7 (58.3)	9 (60.0)	6 (50.0)	8 (53.3)	8 (66.7)
5 days	8 (1.3)	8 (1.3)	6 (75.0)	6 (75.0)	6 (75.0)	4 (50.0)	5 (62.5)	4 (50.0)	5 (62.5)	6 (75.0)	5 (62.5)	5 (62.5)
6 days	1 (0.2)	4 (0.7)	0 (0.0)	3 (75.0)	0 (0.0)	2 (50.0)	0 (0.0)	2 (50.0)	0 (0.0)	4 (100.0)	0 (0.0)	3 (75.0)
7 days	4 (0.7)	4 (0.7)	2 (0.6)	3 (75.0)	1 (25.0)	3 (75.0)	1 (25.0)	3 (75.0)	1 (25.0)	3 (75.0)	1 (25.0)	3 (75.0)
Healthy dietary intake – considering the past 7 days												
**Vegetables**												
0 days	5 (0.8)	9 (2.3)	3 (60.0)	9 (69.2)	4 (80.0)	9 (69.2)	3 (60.0)	8 (61.5)	3 (60.0)	10 (76.9)	3 (60.0)	8 (61.5)
1 day	23 (3.8)	17 (2.8)	15 (65.2)	8 (47.1)	14 (60.9)	7 (41.2)	17 (73.9)	9 (52.9)	13 (56.5)	10 (58.8)	14 (60.9)	7 (41.2)
2 days	34 (5.7)	44 (7.3)	25 (73.5)	26 (59.1)	27 (79.4)	29 (65.9)	28 (82.4)	31 (70.5)	26 (76.5)	29 (65.9)	25 (73.5)	25 (56.8)
3 days	61 (10.2)	41 (6.8)	39 (63.9)	26 (63.4)	42 (68.9)	22 (53.7)	51 (83.6)	22 (53.7)	42 (68.9)	29 (70.7)	39 (63.9)	24 (58.5)
4 days	54 (9.0)	71 (11.8)	31 (57.4)	48 (67.6)	39 (72.2)	48 (67.6)	38 (70.4)	44 (62.0)	31 (57.4)	45 (63.4)	31 (57.4)	47 (66.2)
5 days	87 (14.5)	75 (12.5)	50 (57.5)	48 (64.0)	52 (59.8)	47 (62.7)	54 (62.1)	45 (60.0)	44 (50.6)	52 (69.3)	38 (43.7)	46 (61.3)
6 days	60 (10.0)	80 (13.3)	32 (53.3)	48 (60.0)	35 (58.3)	45 (56.3)	37 (61.7)	54 (67.5)	30 (50.0)	46 (57.5)	29 (48.3)	42 (52.5)
7 days	276 (46.0)	259 (43.2)	123 (44.6)	126 (48.6)	137 (49.6)	107 (41.3)	168 (60.9)	123 (47.5)	133 (48.2)	118 (45.6)	121 (43.8)	105 (40.5)
**Fruits**												
0 days	44 (7.3)	36 (6.0)	30 (68.2)	21 (58.3)	35 (79.5)	23 (63.9)	32 (72.7)	23 (63.9)	30 (68.2)	20 (55.6)	30 (68.2)	20 (55.6)
1 day	43 (7.2)	39 (6.5)	21 (48.8)	26 (66.7)	25 (58.1)	21 (53.8)	31 (72.1)	25 (64.1)	21 (48.8)	25 (64.1)	20 (46.5)	20 (51.3)
2 days	61 (10.2)	58 (9.7)	43 (70.5)	39 (67.2)	42 (68.9)	35 (60.3)	44 (72.1)	39 (67.2)	40 (65.6)	38 (65.5)	40 (65.6)	37 (63.8)
3 days	55 (9.2)	67 (11.2)	30 (54.5)	42 (62.7)	32 (58.2)	36 (53.7)	34 (61.8)	40 (59.7)	32 (58.2)	36 (53.7)	24 (43.6)	35 (52.2)
4 days	67 (11.2)	70 (11.7)	35 (52.2)	40 (57.1)	38 (56.7)	40 (57.1)	46 (68.7)	35 (50.0)	39 (58.2)	41 (58.6)	38 (56.7)	36 (51.4)
5 days	76 (12.7)	75 (12.5)	42 (55.3)	33 (44.0)	49 (64.5)	34 (45.3)	48 (63.2)	43 (57.3)	40 (52.6)	39 (52.0)	36 (47.4)	37 (49.3)
6 days	47 (7.8)	56 (9.3)	21 (44.7)	36 (64.3)	26 (55.3)	31 (55.4)	32 (68.1)	34 (60.7)	25 (53.2)	37 (66.1)	21 (44.7)	32 (57.1)
7 days	207 (34.5)	199 (33.2)	96 (46.4)	102 (51.3)	103 (49.8)	94 (47.2)	129 (62.3)	97 (48.7)	95 (45.9)	103 (51.8)	91 (44.0)	87 (43.7)
Sedentary behavior indicators												
**Physical activity practice?**												
Yes	341 (56.8)	384 (64.0)	142 (41.6)	204 (53.1)	172 (50.4)	176 (45.8)	201 (58.9)	187 (48.7)	155 (45.5)	194 (50.5)	142 (41.6)	204 (53.1)
No	259 (43.2)	216 (36.0)	176 (68.0)	135 (62.5)	178 (68.7)	138 (63.9)	195 (75.3)	149 (69.0)	167 (64.5)	145 (67.1)	176 (68.0)	135 (62.5)
**Physical activity weekly frequency**												
1 day	12 (2.0)	12 (2.0)	3 (25.0)	8 (66.7)	3 (25.0)	9 (75.0)	7 (58.3)	8 (66.7)	4 (33.3)	7 (58.3)	2 (16.7)	7 (58.3)
2 days	73 (12.2)	65 (10.8)	33 (45.2)	37 (56.9)	40 (54.8)	32 (49.2)	45 (61.6)	30 (46.2)	40 (54.8)	37 (56.9)	33 (45.2)	30 (46.2)
3 days	101 (16.8)	123 (20.5)	44 (43.6)	67 (54.5)	56 (55.4)	62 (50.4)	58 (57.4)	63 (51.2)	46 (45.5)	65 (52.8)	44 (43.6)	58 (47.2)
4 days	48 (8.0)	63 (10.5)	21 (43.8)	34 (54.0)	22 (45.8)	20 (31.7)	31 (64.6)	29 (46.0)	20 (41.7)	29 (46.0)	15 (31.3)	23 (36.5)
5 days or more	98 (16.3)	117 (19.5)	36 (36.7)	55 (54.0)	45 (45.9)	51 (43.6)	53 (54.1)	53 (45.3)	40 (40.8)	53 (45.3)	33 (33.7)	46 (39.3)
Unknown	9 (1.5)	4 (0.7)	5 (55.6)	3 (75.0)	6 (66.7)	2 (50.0)	7 (77.8)	4 (100.0)	5 (55.6)	3 (75.0)	6 (66.7)	3 (75.0)
Not physically active	259 (43.2)	216 (36.0)	176 (68.0)	135 (62.5)	178 (68.7)	138 (63.9)	195 (75.3)	149 (69.0)	167 (64.5)	145 (67.1)	167 (64.5)	137 (63.4)
**Television watching time**												
Less than 1 h/day	187 (31.2)	228 (38.0)	95 (50.8)	124 (54.4)	114 (61.0)	122 (53.5)	117 (62.6)	119 (52.2)	96 (51.3)	124 (54.4)	89 (47.6)	106 (46.5)
1h/day	125 20.8)	110 (18.3)	59 (47.2)	64 (58.2)	66 (52.8)	50 (45.5)	79 (63.2)	59 (53.6)	70 (56.0)	67 (60.9)	63 (50.4)	54 (49.1)
2h/day	118 (19.7)	120 (20.0)	61 (51.7)	66 (55.0)	62 (52.5)	62 (51.7)	83 (70.3)	69 (57.5)	66 (55.9)	69 (57.5)	59 (50.0)	61 (50.8)
3h/day	72 (12.0)	66 (11.0)	42 (58.3)	31 (47.0)	41 (56.9)	34 (51.5)	45 (62.5)	36 (54.5)	37 (51.4)	33 (50.0)	34 (47.2)	33 (50.0)
4h/day	35 (5.8)	28 (4.7)	21 (60.0)	21 (75.0)	24 (68.6)	18 (64.3)	26 (74.3)	21 (75.0)	17 (48.6)	15 (53.6)	19 (54.3)	18 (64.3)
5h/day or more	29 (4.8)	5 (0.8)	18 (62.1)	5 (1.5)	18 (62.1)	3 (1.0)	20 (69.0)	5 (1.5)	16 (55.2)	4 (80.0)	14 (48.3)	4 (80.0)
Unknown/varies by day	34 (5.7)	43 (7.2)	22 (64.7)	28 (65.1)	25 (73.5)	25 (58.1)	26 (76.5)	27 (62.8)	20 (58.8)	27 (62.8)	22 (64.7)	28 (65.1)
**Computer use time**												
Less than 1 h/day	10 (1.7)	0 (0.0)	6 (60.0)	0 (0.0)	6 (60.0)	0 (0.0)	7 (70.0)	0 (0.0)	5 (50.0)	0 (0.0)	6 (60.0)	0 (0.0)
1h/day	0 (0.0)	4 (0.3)	0 (0.0)	4 (100)	0 (0.0)	4 (100)	0 (0.0)	4 (100.0)	0 (0.0)	4 (100.0)	0 (0.0)	4 (100.0)
2h/day	14 (2.3)	25 (4.2)	5 (35.7)	13 (52.0)	4 (28.6)	11 (44.0)	9 (64.3)	13 (52.0)	3 (21.4)	11 (44.0)	4 (28.6)	10 (40.0)
3h/day	30 (5.0)	36 (6.0)	14 (46.7)	21 (58.3)	14 (46.7)	15 (41.7)	15 (50.0)	17 (47.2)	11 (36.7)	19 (52.8)	11 (36.7)	14 (38.9)
4h/day	56 (9.3)	54 (9.1)	23 (41.1)	24 (44.4)	27 (48.2)	27 (50.0)	34 (60.7)	29 (53.7)	22 (39.3)	24 (44.4)	21 (37.5)	22 (40.7)
5h/day or more	474 (79.0)	457 (76.7)	259 (54.6)	264 (57.8)	287 (60.5)	243 (53.2)	321 (67.7)	260 (56.9)	273 (57.6)	267 (58.4)	249 (52.5)	241 (52.7)
Unknown/varies by day	16 (2.7)	20 (3.4)	11 (68.8)	11 (55.0)	12 (75.0)	11 (55.0)	10 (62.5)	11 (55.0)	8 (50.0)	12 (60.0)	11 (68.8)	11 (55.0)
Modifiable risk behaviors – considering the past 30 days												
**Cigarette consumption**												
0 days	562 (93.7)	570 (95.0)	301 (53.6)	322 (56.5)	329 (58.5)	301 (52.8)	376 (66.9)	319 (56.0)	303 (53.9)	321 (56.3)	301 (53.6)	322 (56.5)
1–9 days	16 (2.6)	7 (1.2)	5 (31.3)	5 (71.4)	9 (56.3)	4 (57.1)	7 (43.8)	5 (71.4)	6 (37.5)	7 (100.0)	5 (31.3)	5 (71.4)
≥10 days	22 (3.7)	23 (3.8)	12 (54.5)	12 (52.2)	12 (54.5)	9 (39.1)	13 (59.1)	12 (52.2)	13 (59.1)	11 (47.8)	12 (54.5)	12 (52.2)
**Alcohol consumption**												
0 days	189 (31.5)	188 (31.3)	109 (57.7)	107 (56.9)	115 (60.8)	103 (54.8)	140 (74.1)	111 (59.0)	112 (59.3)	112 (59.6)	109 (57.7)	107 (56.9)
1–2 days	129 (21.5)	154 (25.7)	65 (50.4)	94 (61.0)	70 (54.3)	85 (55.2)	82 (63.6)	95 (61.7)	64 (49.6)	86 (55.8)	65 (50.4)	94 (61.0)
3–9 days	195 (32.5)	194 (32.3)	107 (54.9)	97 (50.0)	119 (61.0)	93 (47.9)	130 (66.7)	97 (50.0)	106 (54.4)	112 (57.7)	107 (54.9)	97 (50.0)
≥10 days	87 (14.5)	64 (10.7)	37 (42.5)	41 (64.1)	46 (52.9)	33 (51.6)	44 (50.6)	33 (51.6)	40 (46.0)	29 (45.3)	37 (42.5)	41 (64.1)
**Number of drinks per drinking day**												
0 drinks	188 (31.3)	183 (30.5)	109 (58.0)	103 (56.3)	115 (61.2)	99 (54.1)	139 (73.9)	106 (57.9)	113 (60.1)	109 (59.6)	102 (54.3)	97 (53.0)
<1 drink	31 (5.2)	38 (6.3)	10 (32.3)	24 (63.2)	14 (45.2)	21 (55.3)	18 (58.1)	21 (55.3)	12 (38.7)	26 (68.4)	14 (45.2)	21 (55.3)
1–2 drinks	186 (31.0)	174 (29.0)	96 (51.6)	99 (56.9)	99 (53.2)	89 (51.1)	123 (66.1)	97 (55.7)	88 (47.3)	84 (48.3)	85 (45.7)	87 (50.0)
3–4 drinks	98 (16.3)	120 (20.0)	45 (45.9)	66 (55.0)	52 (53.1)	64 (53.3)	54 (55.1)	67 (55.8)	53 (54.1)	68 (56.7)	40 (40.8)	59 (49.2)
≥5 drinks	97 (16.2)	85 (14.2)	58 (59.8)	47 (55.3)	70 (72.2)	41 (48.2)	62 (63.9)	45 (52.9)	56 (57.7)	52 (61.2)	59 (60.8)	40 (47.1)
Mental health conditions												
**Depression**												
Normal	321 (53.5)	315 (52.5)	109 (34.0)	123 (39.0)	115 (35.8)	101 (32.1)	172 (53.6)	123 (39.0)	125 (38.9)	124 (39.4)	93 (29.0)	89 (28.3)
Mild/Moderate	186 (31.0)	166 (27.7)	129 (69.4)	116 (69.9)	148 (79.6)	113 (68.1)	141 (75.8)	114 (68.7)	122 (65.6)	114 (68.7)	125 (67.2)	115 (69.3)
Severe/Extremely severe	93 (15.5)	119 (19.8)	80 (86.0)	100 (84.0)	87 (93.5)	100 (84.0)	83 (89.2)	99 (83.2)	75 (80.6)	101 (84.9)	82 (88.2)	100 (84.0)
**Anxiety**												
Normal	326 (54.3)	332 (55.3)	120 (36.8)	150 (45.2)	138 (42.3)	130 (39.2)	184 (56.4)	138 (41.6)	133 (40.8)	144 (43.4)	112 (34.4)	118 (35.5)
Mild/Moderate	147 (24.5)	138 (23.0)	95 (64.6)	86 (62.3)	103 (70.1)	85 (61.6)	100 (68.0)	93 (67.4)	92 (62.6)	91 (65.9)	84 (57.1)	84 (60.9)
Severe/Extremely severe	127 (21.2)	130 (21.7)	103 (81.1)	103 (79.2)	109 (85.8)	99 (76.2)	112 (88.2)	105 (80.8)	97 (76.4)	104 (80.0)	104 (81.9)	102 (78.5)
**Stress**												
Normal	328 (54.7)	315 (52.5)	114 (34.8)	138 (43.8)	128 (39.0)	117 (37.1)	178 (54.3)	129 (41.0)	130 (39.6)	134 (42.5)	99 (30.2)	107 (34.0)
Mild/Moderate	156 (26.0)	170 (28.3)	102 (65.4)	113 (66.5)	114 (73.1)	108 (63.5)	119 (76.3)	114 (67.1)	100 (64.1)	107 (62.9)	100 (64.1)	102 (60.0)
Severe/Extremely severe	116 (19.3)	115 (19.2)	102 (87.9)	88 (76.5)	108 (93.1)	89 (77.4)	99 (85.3)	93 (80.9)	92 (79.3)	98 (85.2)	101 (87.1)	95 (82.6)

**Note:** The table presents descriptive data of the WHOQOL-bref domains by wave of the longitudinal study. Absolute and relative frequencies of low QoL are shown, categorized based on the median of each domain. Values below the median were considered as low QoL. * “HS” is an abbreviation for high school, “HE” for higher education, “TPE” for technical professional education, and “MBA” for Master of Business Administration.

The median QoL scores were calculated separately for each year of data collection (2022 and 2023), according to the WHOQOL-bref domains. The median scores were 64.3 and 57.1 for the physical domain, 66.7 and 62.5 for the psychological domain, 66.7 and 58.3 for the social relationships domain, 65.6 and 65.6 for the environment domain, and 64.3 and 61.3 for the total score, respectively (data in S1 Table). The results of the paired t-test indicated significant reductions (p < 0.05) in the WHOQOL-bref domain scores between the first data collection (2022) and the second (2023). When stratified by sex, both males and females showed significant decreases from 2022 to 2023 (p < 0.05). However, in the physical domain, men did not exhibit a significant difference (p > 0.05) between the two data collection points (Fig 1 and data in S1 Table).

**Fig 1:**
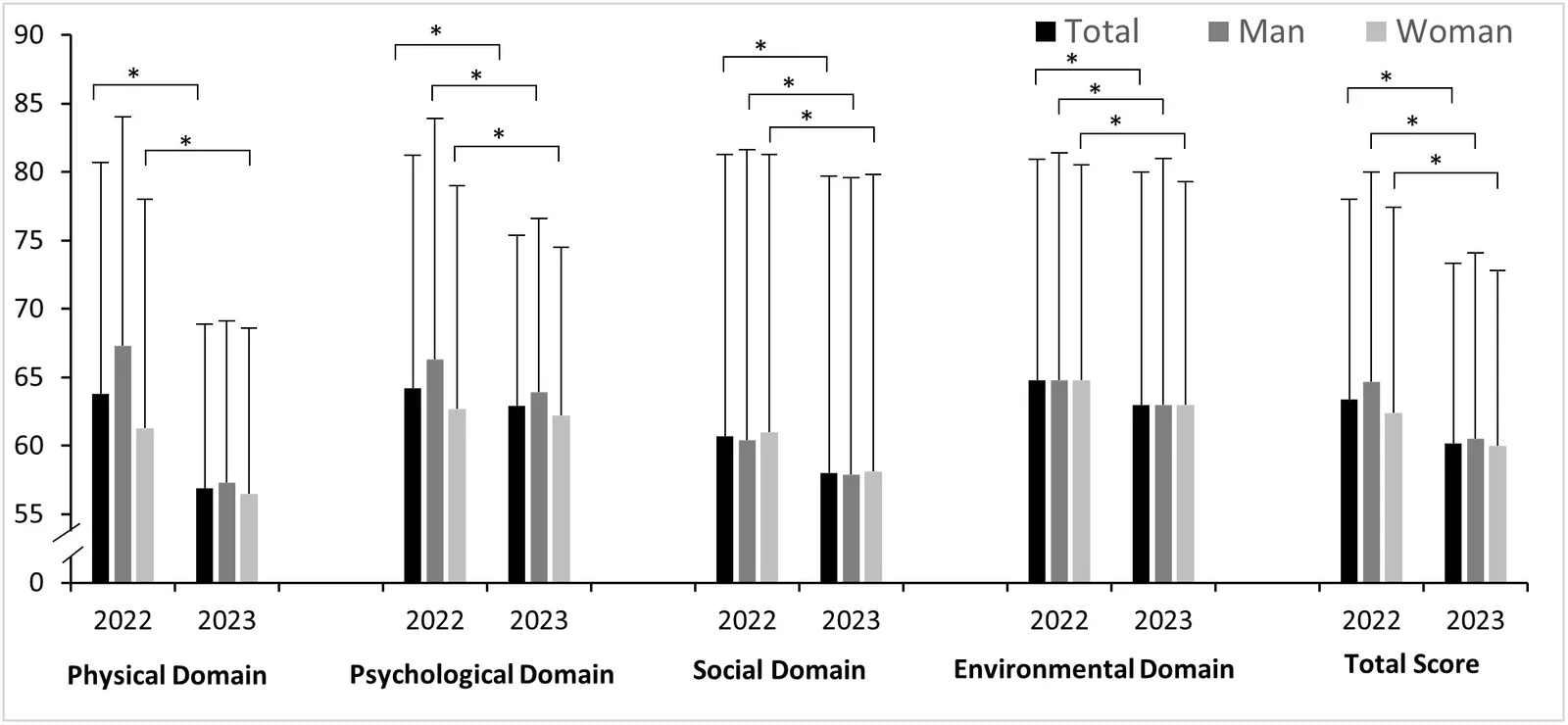
Mean and Standard Deviation by domain of the WHOQOL-bref. Legend: Bar charts displaying mean values and standard deviation for each quality of life domain (Physical, Psychological, Social, and Environmental) and total score, stratified by biological sex (Total, Man, Woman) across two waves of data collection (2022 and 2023). Paired t-test was used to compare measurements between the two years within each group. Asterisks (*) indicate significant differences (p < 0.05). SD, standard deviation.

The bivariate analysis (data in S2 Table) indicated that low QoL, represented by the physical domain, was associated (p < 0.05) with sociodemographic factors (female sex and region), lifestyle variables (regular consumption of ultra-processed foods and fast food, irregular consumption of vegetables/greens and fruits, physical inactivity, and television time of 5 hours or more per day), and mental health variables (depression, anxiety, and stress).

The adjusted analysis by sex, age group, education level, region, and position (Table 2) indicated that female sex is a risk factor for low QoL in the physical (*RRadj* = 1.19; 1.05–1.34; p = 0.005) and psychological (*RRadj* = 1.14; 1.01–1.30; p = 0.045) domains. Having an age of 60 years or older is a protective factor when associated with low QoL (*RRadj* = 0.67; 0.46–0.99; p = 0.043). The North and Northeast regions were associated with low QoL in the social relationships domain (*RRadj* = 1.24; 1.03–1.48; p = 0.023). Another identified risk factor was among professionals assigned as Administrative-technical, in the environmental domain (*RRadj* = 1.27; 1.07–1.51; p = 0.007), compared to teachers. An association (p < 0.05) was observed between regular UPF consumption and irregular fruit consumption with low QoL in the physical (*RRadj* = 1.29; 1.14–1.46; p < 0.001), psychological (*RRadj* = 1.21; 1.08–1.36; p = 0.001), environment domains (*RRadj* = 1.16; 1.04–1.31; p = 0.011), and total score (*RRadj* = 1.19; 1.04–1.36; p = 0.010). Physical inactivity was also strongly associated with low QoL in all WHOQOL-bref domains (Table 2). In this adjusted analysis, the consumption of alcoholic beverages for 10 days or more during the month remained associated with higher scores (*RRadj* = 0.83; 0.69–0.99; p = 0.037), along with the intake of 3–4 glasses/doses per day in the social relationships domain (*RRadj* = 0.86; 0.75–0.99; p = 0.044) and 1–2 glasses/doses per day in the environment domain (*RRadj* = 0.87; 0.77–0.99; p = 0.044). Depression, anxiety, and stress, in mild/moderate and severe/very severe classifications, were associated as risk factors with all domains of the WHOQOL-bref (Table 2).

**Table 2:** GEE-adjusted Estimates for Factors Associated with WHOQOL-bref Domains Among education professionals: A Two-Wave Longitudinal Study in Brazil, 2022–2023 (N = 600).

Variables	Physical Domain – Low QoL		Psychological Domain – Low QoL		Social Relations Domain – Low QoL		Environment Domain – Low QoL		Total Score – Low QoL	
Sociodemographic	RRadj Analysis	*p*	RRadj Analysis	*p*	RRadj Analysis	*p*	RRadj Analysis	*p*	RRadj Analysis	*p*
**Sex**										
Male	1		1		1		–	–	–	–
Female	**1.19 (1.05 - 1.34)**	0.005	**1.14 (1.01 - 1.30)**	0.045	0.98 (0.88 - 1.09)	0.717				
**Age group**										
≤39	1		1		–	–	1		1	
40–59	1.01 (0.90 - 1.13)	0.874	0.89 (0.80 - 1.01)	0.059			1.04 (0.92 - 1.17)	0.570	0.95 (0.84 - 1.09)	0.954
≥60	0.71 (0.48 - 1.04)	0.076	**0.67 (0.46 - 0.99)**	0.043			0.75 (0.50 - 1.12)	0.162	0.76 (0.52 - 1.12)	0.764
**Educational level**										
Doctarate/Postdoctoral	–	–	1		1		1		1	
Master’s degree			1.10 (0.91 - 1.33)	0.318	0.99 (0.85 - 1.16)	0.882	0.93 (0.77 - 1.13)	0.459	1.05 (0.84 - 1.31)	0.659
Lato Sensu/MBA			**1.26 (1.02 - 1.55)**	0.030	1.14 (0.96 - 1.36)	0.130	1.06 (0.87 - 1.31)	0.565	1.18 (0.93 - 1.51)	0.172
HS/HE/TPE*			1.22 (0.93 - 1.60)	0.157	1.06 (0.83 - 1.36)	0.645	1.01 (0.75 - 1.35)	0.949	1.09 (0.79 - 1.51)	0.600
**Region**										
South	1		1		1		1		1	
Southeast	1.05 (0.88 - 1.25)	0.628	1.07 (0.89 - 1.30)	0.464	1.03 (0.88 - 1.20)	0.759	1.06 (0.88 - 1.29)	0.530	1.05 (0.85 - 1.30)	0.641
Midwest	0.96 (0.80 - 1.15)	0.627	0.95 (0.79 - 1.16)	0.625	0.95 (0.81 - 1.12)	0.567	1.02 (0.83 - 1.24)	0.862	1.00 (0.81 - 1.24)	0.991
North and Northeast	1.16 (0.98 - 1.37)	0.095	1.13 (0.95 - 1.35)	0.179	1.07 (0.92 - 1.24)	0.409	**1.24 (1.03 - 1.48)**	0.023	1.15 (0.94 - 1.40)	0.186
**Position**										
Teacher			1		1		1		1	
Administrative-technical staff	–	–	1.05 (0.90 - 1.24)	0.524	1.03 (0.90 - 1.19)	0.680	**1.27 (1.07 - 1.51)**	0.007	1.07 (0.89 - 1.28)	0.507
Lifestyle Habits										
Unhealthy dietary intake – considering the past 7 days										
**UPF**										
0–4 days	1		1		–	–	1		1	
5–7 days	**1.29 (1.14 - 1.46)**	<0.001	**1.21 (1.08 - 1.36)**	0.001			**1.16 (1.04 - 1.31)**	0.011	**1.19 (1.04 - 1.36)**	0.010
***Fast food*:**										
0–4 days	1		–	–	–	–	–	–	1	
5–7 days	**1.34 (1.05 - 1.72)**	0.021							1.30 (1.00 - 1.68)	0.053
Healthy dietary intake – considering the past 7 days										
**Vegetables**										
5–7 days	1		1		1		1		1	
0–4 days	**1.19 (1.07 - 1.33)**	0.001	**1.21 (1.10 - 1.33)**	<0.001	**1.12 (1.02 - 1.24)**	0.016	**1.13 (1.02 - 1.26)**	0.016	**1.21 (1.08 - 1.36)**	0.001
**Fruits**										
5–7 days	1		1		1		1		1	
0–4 days	**1.17 (1.06 - 1.30)**	0.003	**1.13 (1.03 - 1.23)**	0.011	1.07 (0.98 - 1.17)	0.154	**1.11 (1.01 - 1.22)**	0.028	**1.13 (1.02 - 1.24)**	0.019
Sedentary behavior indicators										
**Physical activity practice?**										
Yes	1		1		1		1		1	
No	**1.36 (1.22 - 1.51)**	<0.001	**1.30 (1.18 - 1.43)**	<0.001	**1.29 (1.18 - 1.42)**	<0.001	**1.21 (1.10 - 1.33)**	<0.001	**1.37 (1.23 - 1.52)**	<0.001
**Physical activity weekly frequency**										
≥5 days	1		1		1		1		1	1
3-4 days	1.13 (0.94 - 1.36)	0.201	1.01 (0.85 - 1.19)	0.958	1.09 (0.92 - 1.28)	0.318	1.02 (0.86 - 1.22)	0.822	1.04 (0.85 - 1.27)	0.701
1-2 days	1.15 (0.94 - 1.42)	0.183	1.07 (0.88 - 1.30)	0.519	1.13 (0.93 - 1.37)	0.221	1.08 (0.88 - 1.32)	0.465	1.10 (0.88 - 1.37)	0.390
0 days	**1.50 (1.27 - 1.78)**	<0.001	**1.33 (1.14 - 1.55)**	<0.001	**1.40 (1.21 - 1.63)**	<0.001	**1.26 (1.07 - 1.48)**	0.012	**1.45 (1.21 - 1.75)**	<0.001
**Television watching time**										
≤2 hours	1		1		1		–	–	1	
3–4 hours	1.12 (0.97 - 1.29)	0.129	1.14 (1.00 - 1.30)	0.058	1.12 (0.99 - 1.27)	0.077			1.16 (1.01 - 1.34)	0.048
≥5 hours	**1.33 (1.09 - 1.63)**	0.005	1.15 (0.91 - 1.44)	0.245	**1.32 (1.10 - 1.59)**	0.003			1.05 (0.81 - 1.37)	0.702
**Computer use time**										
≤2 hours	**–**	–	–	–	**–**	–	1		–	–
3–4 hours							0.95 (0.73 - 1.23)	0.678		
≥5 hours							1.09 (0.85 - 1.39)	0.488		
Modifiable risk behaviors – Considering the past 30 days										
**Alcohol consumption**										
0 days	–	–	1		1		1		1	
1–2 days			0.93 (0.81 - 1.06)	0.275	0.95 (0.84 - 1.07)	0.352	0.92 (0.81 - 1.04)	0.185	0.99 (0.86 - 1.14)	0.879
3–9 days			1.01 (0.89 - 1.16)	0.844	0.90 (0.79 - 1.02)	0.089	1.01 (0.89 - 1.14)	0.916	0.95 (0.81 - 1.10)	0.468
≥10 days			1.01 (0.85 - 1.20)	0.932	**0.83 (0.69 - 0.99)**	0.037	0.86 (0.71 - 1.04)	0.114	0.82 (0.67 - 1.02)	0.071
**Number of drinks per drinking day**										
0 drinks	–	–	1		1		1		1	
<1 drink			1.00 (0.81 - 1.22)	0.959	0.92 (0.75 - 1.15)	0.473	0.99 (0.81 - 1.20)	0.879	1.06 (0.87 - 1.29)	0.540
1–2 drinks			0.92 (0.81 - 1.06)	0.250	0.96 (0.86 - 1.09)	0.541	**0.87 (0.77 - 0.99)**	0.044	0.96 (0.83 - 1.12)	0.596
3–4 drinks			1.00 (0.87 - 1.16)	0.997	**0.86 (0.75 - 0.99)**	0.044	0.98 (0.85 - 1.12)	0.736	0.85 (0.71 - 1.02)	0.080
≥5 drinks			1.07 (0.92 - 1.26)	0.385	0.90 (0.77 - 1.05)	0.174	0.98 (0.85 - 1.12)	0.813	0.99 (0.83 - 1.19)	0.930
Mental health conditions										
**Depression**										
Normal	1		1		1		1		1	
Mild/Moderate	**1.86 (1.65 - 2.10)**	<0.001	**1.92 (1.69 - 2.18)**	<0.001	**1.47 (1.31 - 1.64)**	<0.001	**1.52 (1.36 - 1.70)**	<0.001	**2.05 (1.77 - 2.37)**	<0.001
Severe/Extremely severe	**2.31 (2.05 - 2.60)**	<0.001	**2.29 (2.01 - 2.61)**	<0.001	**1.75 (1.57 - 1.95)**	<0.001	**1.91 (1.69 - 2.15)**	<0.001	**2.61 (2.25 - 3.02)**	<0.001
**Anxiety**										
Normal	1		1		1		1		1	
Mild/Moderate	**1.49 (1.31 - 1.70)**	<0.001	**1.41 (1.24 - 1.60)**	<0.001	**1.33 (1.19 - 1.49)**	<0.001	**1.38 (1.22 - 1.56)**	<0.001	**1.51 (1.31 - 1.73)**	<0.001
Severe/Extremely severe	**1.85 (1.64 - 2.07)**	<0.001	**1.71 (1.52 - 1.92)**	<0.001	**1.66 (1.50 - 1.83)**	<0.001	**1.69 (1.50 - 1.91)**	<0.001	**1.97 (1.72 - 2.25)**	<0.001
**Stress**										
Normal	1		1		1		1		1	
Mild/Moderate	**1.67 (1.47 - 1.90)**	<0.001	**1.59 (1.41 - 1.79)**	<0.001	**1.43 (1.28 - 1.59)**	<0.001	**1.38 (1.24 - 1.55)**	<0.001	**1.68 (1.46 - 1.93)**	<0.001
Severe/Extremely severe	**2.03 (1.80 - 2.28)**	<0.001	**1.94 (1.72 - 2.19)**	<0.001	**1.68 (1.52 - 1.86)**	<0.001	**1.78 (1.58 - 2.00)**	<0.001	**2.30 (2.02 - 2.63)**	<0.001

**Note:** Adjusted Relative Risk (RRadj) estimates were obtained using Generalized Estimating Equations (GEE) models adjusted for sociodemographic variables: sex, age group, educational level, region, and position. Only variables with *p* < 0.20 in the bivariate analysis (data in S2 Table) were included in this analysis. * “HS” is an abbreviation for high school, “HE” for higher education, “TPE” for technical professional education, and “MBA” for Master of Business Administration.

## Discussion

This longitudinal study aimed to assess, through a longitudinal design, the association of lifestyle variables, modifiable risk behaviors, and mental health with QoL among of the education professionals. Our main findings include a reduction in QoL across all domains in the overall sample from 2022 to 2023, and a risk of low QoL associated with the following variables: female sex (physical and psychological domains), educational level of specialization or MBA (psychological domain), North and Northeast regions, occupation as Administrative-technical staff (social relationships domain), regular consumption of UPF (physical, psychological, environmental domains, and overall score), low intake of legumes, vegetables, and fruits, physical inactivity (all domains), television time of 5 hours or more (physical and social relationships domains), and mild/moderate and severe/extremely severe mental health conditions, including depression, anxiety, and stress, in all domains. On the other hand, some variables were associated with higher QoL scores, such as being 60 years of age or older in the psychological domain, and alcohol consumption in the social relationships domain. The results obtained reinforce the multifactorial complexity of the determinants of QoL, highlighting the need for integrated strategies to promote the well-being of this population.

The scientific literature presents relevant and similar evidence of associations between female sex and low QoL [51–54], especially in the physical and psychological domains, which assess indicators of physical health and emotional aspects, respectively [55,56]. However, several interconnected factors contribute to the higher prevalence of lower QoL among women, including biological factors that lead to hormonal changes (pregnancy, polycystic ovary syndrome, menopause, among others) [57,58], social factors such as domestic violence, gender discrimination, and the overload of tasks that have historically been performed by women, resulting in an exhausting double burden to balance work and family [59,60]. Additionally, psychological factors [61,62] are also possible contributors, since symptoms of depression, anxiety, stress, low self-esteem, and body dissatisfaction are more prevalent among women compared to men, especially among women working in the education sector [63]. These findings underscore the pronounced gender-based disparities in Quality of Life (QoL) and highlight the necessity for further research to elucidate the underlying determinants affecting this population.

The level of education, particularly specialization/MBA, constituted a risk factor for low QoL in the psychological domain in our study, compared with the doctoral level. Although there is no solid and in-depth evidence in the literature regarding the relationship between each level of education and QoL, some studies indicate that education positively influences QoL, as it increases the likelihood of obtaining better-paid jobs and improved working conditions [64,65]. Therefore, the strength of this relationship may be linked to broader social, economic, and individual factors [66,67]. TAs professionals, for example, were at greater risk of low QoL over time in the environmental domain, which includes aspects such as physical safety, financial resources, access to health services, leisure, transportation, and exposure to pollution and noise [65,68]. Jobs with lower autonomy, greater physical demands, and unhealthy environments tend to negatively affect these aspects, whereas positions with better conditions and greater control favor higher environmental scores [68]. The findings of this study are consistent with evidence indicating that pressure for academic progression and high occupational demands associated with higher qualification levels may negatively influence QoL [69, 70]. Education professionals residing in the North and Northeast regions of Brazil showed low QoL in the environmental domain, reinforcing the need for future studies to assess the specificities and characteristics of each region, analyzing their social and cultural aspects to contribute to targeted interventions [71,72].

Cross-sectional and cohort studies have shown consistent associations between the consumption of UPF, including fast food, and low QoL, as well as an increased risk of physical and mental health problems in adult populations [17,18,73,74]. A few large multinational companies control the production of UPF, applying strategic marketing and thereby generating high profitability, replacing other types of foods, and promoting dietary patterns associated with chronic diseases and obesity [20,75]. Digital media, through sponsorships, invest in advertising campaigns focused on intensifying the large-scale sale of UPF in most countries [76]. The UPF-based dietary pattern is quite common among professionals, especially those in the education sector, due to demanding routines, stress, and the imbalance between professional and personal life [74]. Similarly, our study indicated that regular consumption of UPF is associated with low QoL in the physical, psychological, and environmental domains, as well as in the total WHOQOL-bref score, with regular consumption of fast food increasing the risk of low QoL in the physical domain. Therefore, the availability and accessibility of minimally processed foods are recommended, promoted through effective public and institutional policies, with notable involvement of media and mass communication channels, to encourage the reduction of UPF consumption, particularly amongst education professionals [76]. Another area of public policy currently under discussion focuses on promoting healthy food environments through subsidies aimed at making fresh and minimally processed foods, such as fruits and vegetables, more affordable, alongside restrictions on the sale of ultra-processed foods in educational institutions and their surrounding areas.

The regular consumption of healthy foods, such as fruits and vegetables, is recommended. Irregular consumption was associated with poorer QoL in physical, psychological, social, and environmental aspects. To promote well-being and prevent chronic diseases, the World Health Organization (WHO) recommends the daily consumption of at least 400 grams, or approximately five servings, of fruits and vegetables [77]. Additionally, it emphasizes the importance of the regular intake of legumes, such as beans, lentils, and peas, as part of a healthy, plant-based diet [78,79]. This guidance is widely supported by studies highlighting its benefits for physical and mental health, including the reduction of the risk of cardiovascular diseases, cancer, hypertension, and overall mortality [80–82]. Our findings align with prospective studies and meta-analyses in adults that support higher fruit and vegetable intake to improve QoL [83–86]. Therefore, increasing the consumption of these foods is a key, evidence-based strategy for improving physical and mental well-being, while supporting education professionals about healthy dietary habits.

A healthy lifestyle needs to incorporate physical activity as a key factor in promoting physical and mental well-being, with a strong association with improved QoL across various populations and in all domains [87,88]. Physical activity significantly improves functional capacity, mood, independence, and resilience, while proportionally reducing symptoms of anxiety and depression [89,90]. Social interaction during physical activity also contributes to mental health [89]. The frequency of moderate-to-vigorous activities, as well as regular exercise of at least three times per week, are consistently associated with higher QoL scores [91,92]. Although our study did not find a significant association between weekly frequency of physical activity and low QoL, not engaging in any physical activity during the week demonstrated a strong risk of low QoL across all dimensions.

Regarding sedentary habits, excessive time spent watching television is associated with poorer QoL in physical and social relationship aspects. Some studies emphasize the health-related effects of this behavior, such as obesity, diabetes, depression, and increased loneliness [93–95]. In line with the literature indicating that computer use is less consistently associated with health problems [96,97], our data did not show significant associations with excessive use; however, excessive screen time in general is frequently studied due to its potential to generate a range of adverse consequences [98,99]. Therefore, the situation in Brazil calls for public education policies that support education professionals in adopting and promoting active lifestyles, thereby combating the effects of excessive screen time. While there are public policies aimed at students, there are gaps and structural challenges regarding direct, specific support for the health and physical activity routines of the education professionals themselves.

Depression, anxiety, and stress are consistently associated with poorer QoL across multiple domains (physical, psychological, social relationships, environment, and total score). This can lead to somatic symptoms (fatigue, insomnia, pain) and emotional symptoms (sadness, hopelessness), contributing to functional limitations, social isolation, and lower life satisfaction [100,101]. Similar to the literature, our study highlighted the impact of mental health across all domains, indicating that symptoms of depression, anxiety, and stress are associated with low QoL, even after adjustment for sociodemographic factors. Studies conducted in Iran and Spain, respectively [102,103] indicated that high levels of perceived stress are linked to poorer QoL, especially in crisis situations such as the COVID-19 pandemic, further exacerbating symptoms of anxiety and depression and negatively impacting overall well-being. Although anxiety and depression contribute to low QoL, some studies investigate the bidirectional effect, in which low QoL also increases negative mental health symptoms [104,105], highlighting the need for interventions, such as psychotherapy, to achieve parallel improvements in QoL, especially in the psychological and physical domains [106,107].

Healthy and active aging is positively associated with a better perception of QoL among older adults [108] and this association can be explained in various ways in the literature. Maintaining independence, functionality, and active participation in daily, social, cultural, and economic activities, even with limitations, is linked to greater satisfaction and well-being [108–110]. Additionally, resilience, social support, and healthy lifestyle habits—such as a balanced diet, regular physical activity, adequate sleep, etc.—are important mediators for older adults to overcome challenges while remaining engaged in daily activities [22,108,111]. Our study indicates that being 60 years of age or older is a protective factor for QoL. A possible explanation is that professionals in this age group possess greater maturity, mastery of administrative and academic tasks, confidence in performing their duties, and higher job satisfaction, resulting in lower occupational stress and reduced physical and psychological work-related burden [112,113]. This age group also tends to exhibit greater emotional control and a stronger sense of institutional recognition, which promotes a better balance between professional and personal life [114].

Frequent or excessive alcohol consumption has been evaluated in several studies and has been associated with poorer QoL, including the social domain [115–117]. Paradoxically, our study indicated an unexpected inverse association between alcohol consumption on 10 days in the previous month and low QoL in the social relationships domain, a finding that warrants careful interpretation [115,118]. This finding was isolated, modest in magnitude, and was not accompanied by a consistent gradient across categories of alcohol consumption frequency or quantity. Therefore, it should not be interpreted as evidence that alcohol consumption improves QoL. A possible explanation lies in the social attribution model, which suggests that alcohol reduces fear of social stressors and improves the quality of interpersonal interactions, offering social rewards that may be particularly valuable for individuals who find social situations challenging [118]. However, it is important to highlight that not all expectancies surrounding alcohol use carry the same risk, as individuals with stronger sociability expectancies, that is, the belief that alcohol facilitates social interaction, have higher odds of engaging in heavy drinking episodes and experiencing alcohol-related problems, whereas stronger tension reduction expectancies were associated with lower odds of these outcomes [119]. Although alcohol may increase social inclusion and reduce momentary loneliness in certain contexts [115,120], these practices may become excessive and chronic, leading to long-term harm to health and QoL [117,121]. Our findings represent a call to action, highlighting the need for alternative strategies, such as social skills training and community-based interventions, to improve social functioning without relying on alcohol. Since alcohol consumption and QoL were assessed simultaneously at each time point, the temporal direction of the association cannot be established. Alcohol consumption may function, in part, as a marker of participation in social events rather than as a cause of better social quality of life. Future studies should characterize drinking patterns and social contexts more precisely and examine whether alcohol consumption prospectively predicts changes in QoL.

Despite the relevance of our findings, particularly regarding factors such as biological sex, healthy and unhealthy dietary intake, physical activity, television time, alcohol consumption, and mental health issues, it is necessary to address some limitations. The responses in this longitudinal study are self-reported, and we cannot rule out the possibility of recall bias and inaccuracies in providing reliable information, particularly regarding dietary habits and modifiable risk behaviors, which assess the previous 7 and 30 days, respectively, prior to data collection. For future studies, it would be feasible to explore regional issues in greater depth to enable comparisons across Brazilian regions, as well as to more broadly assess physical activity, including which activities or sports are practiced, given that this variable proved to be a determinant of QoL over time. Longitudinal investigations over longer follow-up periods are also warranted, since habits and quality of life may require more than one year to show substantial changes. The recruitment strategy is also a source of potential selection bias: participation depended on having an institutional email address publicly available on the institution’s website and voluntarily responding to an online invitation, which may have favored professionals who were more engaged with health-related issues or who had better work conditions. It is essential to exercise caution when generalizing these results to the entire Brazilian population, as the study population consists of public education professionals, with specific characteristics and a particular career plan. Despite the aforementioned limitations, we believe they do not compromise the validity of the conclusions drawn.

The strength of this study lies in its longitudinal approach, allowing for the assessment of sociodemographic variables, lifestyle habits, modifiable risk behaviors, and mental health over time, including all regions of Brazil. In practice, the health of education professionals must be taken seriously and incorporated into educational policies. Workplace health promotion could include opportunities for physical activity as part of the work routine, a school environment that offers fresh and minimally processed foods, occasional breaks to step away from screens, as well as ongoing and confidential psychological support, along with realistic workloads and requirements that do not compromise the physical and mental health of these professionals. Additionally, the findings contribute to the development of knowledge related to epidemiological data.

## Conclusion

The highest prevalence of low QoL is observed among women, compared to men. Regular consumption of UPF, irregular consumption of healthy foods, physical inactivity, prolonged television exposure, and symptoms of depression, anxiety, and stress are risk factors for low QoL among education professionals. On the other hand, being 60 years of age or older was a protective factor against low QoL, and alcohol consumption on 10 or more days per month was also associated with higher scores in the social domain. Therefore, improving modifiable healthy lifestyle habits is the way to enhance QoL, providing a harmonious and productive work environment and contributing to the collective well-being and productivity of education professionals. In this context, psychological support acts as a facilitating factor in the adoption of healthy habits, promoting beneficial and sustainable long-term health outcomes.

## Supporting information

S1 Table Mean ± standard deviation, median, and paired t-test results by biological sex across two waves of data collection among education professionals (2022–2023), Brazil (N = 600). Note: Data are presented as mean ± standard deviation (SD) and median values for each domain (Physical, Psychological, Social Relationships, and Environment) stratified by biological sex (female, male, and total sample). The paired t-test compared measurements between 2022 (baseline) and 2023 (follow-up). The total score represents the overall quality of life measure across all domains. Statistical significance was set at p < 0.05. Values highlighted in bold indicate significant differences between the two waves of data collection within each group.
(DOCX)

S2 Table Bivariate analysis of sociodemographic, lifestyle, and mental health factors associated with low Quality of Life (QoL) among education professionals across two waves of data collection (2022–2023), Brazil (N = 600). Note: The table presents the results of the bivariate analysis between the independent variables and the outcome of low QoL, defined based on the median of each WHOQOL-bref domain. Scores below the median were considered as low QoL. Estimates were obtained using Generalized Estimating Equations (GEE) models. * “HS” is an abbreviation for high school, “HE” for higher education, “TPE” for technical professional education, and “MBA” for Master of Business Administration.
(DOCX)
