# Supplementary material for: What is the cost of dedication to teaching? A school-based longitudinal quantitative study on habits and quality of life
Source: PLoS One. 2026 Sep 29;21(9):e0359001. doi: 10.1371/journal.pone.0359001 (PMC13623129; doi:10.1371/journal.pone.0359001)
Supplement: Supplementary file 1 [file pone.0359001.s001.docx]

**What is the Cost of Dedication to Teaching? A prospective, school-based longitudinal panel study on Habits and Quality of Life**

# Supplementary materials

**Table 1.** Mean ± standard deviation, median, and paired t-test results by biological sex across two waves of data collection among education professionals (2022–2023), Brazil (N = 600).

| **Domain** | **Sex** | **Mean 2022 ± SD** | **Mean 2023 ± SD** | **Median 2022** | **Median 2023** | **t** | ***p*-value** |
| --- | --- | --- | --- | --- | --- | --- | --- |
| Physical | Female | 61.3 ± 16.7 | 56.5 ± 12.1 | 60.7 | 57.1 | 5.04 | <0.001 |
|  | Male | 67.3 ± 16.7 | 57.3 ± 11.8 | 67.9 | 57.1 | 9.57 | 0.478 |
|  | Total | 63.8 ± 16.9 | 56.9 ± 12.0 | 64.3 | 57.1 | 9.76 | <0.001 |
| Psychological | Female | 62.7 ± 16.3 | 62.2 ± 12.3 | 62.5 | 62.5 | 0.71 | 0.008 |
|  | Male | 66.3 ± 17.6 | 63.9 ± 12.7 | 66.7 | 66.7 | 2.84 | 0.008 |
|  | Total | 64.2 ± 17.0 | 62.9 ± 12.5 | 66.7 | 62.5 | 2.45 | 0.015 |
| Social Relationships | Female | 61.0 ± 20.3 | 58.1 ± 21.7 | 66.7 | 58.3 | 2.66 | <0.001 |
|  | Male | 60.4 ± 21.2 | 57.9 ± 21.8 | 60.8 | 58.3 | 2.09 | <0.001 |
|  | Total | 60.7 ± 20.6 | 58.0 ± 21.7 | 66.7 | 58.3 | 2.38 | 0.001 |
| Environment | Female | 64.8 ± 15.7 | 63.0 ± 16.3 | 65.6 | 62.5 | 2.69 | 0.005 |
|  | Male | 64.8 ± 16.6 | 63.0 ± 18.0 | 65.6 | 65.6 | 2.09 | 0.038 |
|  | Total | 64.8 ± 16.1 | 63.0 ± 17.0 | 65.6 | 65.6 | 3.40 | 0.001 |
| Total Score | Female | 62.4 ± 14.1 | 60.0 ± 12.8 | 63.4 | 60.9 | 4.30 | 0.037 |
|  | Male | 64.7 ± 15.3 | 60.5 ± 13.6 | 65.0 | 61.4 | 5.87 | <0.001 |
|  | Total | 63.4 ± 14.6 | 60.2 ± 13.1 | 64.3 | 61.3 | 7.09 | <0.001 |

**Note:** Data are presented as mean ± standard deviation (SD) and median values for each domain (Physical, Psychological, Social Relationships, and Environment) stratified by biological sex (female, male, and total sample). The paired t-test compared measurements between 2022 (baseline) and 2023 (follow-up). The total score represents the overall quality of life measure across all domains. Statistical significance was set at p < 0.05. Values highlighted in bold indicate significant differences between the two waves of data collection within each group.
