# Supplementary material for: What is the cost of dedication to teaching? A school-based longitudinal quantitative study on habits and quality of life
Source: PLoS One. 2026 Sep 29;21(9):e0359001. doi: 10.1371/journal.pone.0359001 (PMC13623129; doi:10.1371/journal.pone.0359001)
Supplement: Supplementary file 2 [file pone.0359001.s002.docx]

**What is the Cost of Dedication to Teaching? A prospective, school-based longitudinal panel study on Habits and Quality of Life**

# Supplementary materials

# Table 2. Bivariate analysis of sociodemographic, lifestyle, and mental health factors associated with low Quality of Life (QoL) among education professionals across two waves of data collection (2022–2023), Brazil (N = 600).

| **Variables** | **Physical Domain - Low QoL** | | **Psychological Domain - Low QoL** | | **Social Relations Domain - Low QoL** | | **Environment Domain - Low QoL** | | **Total Score - Low QoL** | |
| --- | --- | --- | --- | --- | --- | --- | --- | --- | --- | --- |
| **Sociodemographic Issues** | RR Analysis | *p* | RR Analysis | *p* | RR Analysis | *p* | RR Analysis | *p* | RR Analysis | *p* |
| **Sex** |  |  |  |  |  |  |  |  |  |  |
| Male | 1 |  | 1 |  | 1 |  | 1 |  | 1 |  |
| Female | **1.19 (1.06 - 1.35)** | 0.004 | **1.17 (1.02 - 1.33)** | 0.021 | 0.99 (0.89 - 1.10) | 0.839 | 1.05 (0.92 - 1.19) | 0.485 | 1.06 (0.92 - 1.22) | 0.416 |
| **Age group** |  |  |  |  |  |  |  |  |  |  |
| ≤39 | 1 |  | 1 |  | 1 |  | 1 |  | 1 |  |
| 40–59 | 1.01 (0.90 - 1.13) | 0.893 | **0.87 (0.77 - 0.98)** | 0.018 | 0.97 (0.87 - 1.08) | 0.559 | 1.00 (0.88 - 1.13) | 0.994 | 0.93 (0.82 - 1.06) | 0.093 |
| ≥60 | 0.69 (0.47 - 1.02) | 0.060 | **0.63 (0.43 - 0.93)** | 0.021 | 0.87 (0.66 - 1.14) | 0.311 | 0.68 (0.45 - 1.02) | 0.063 | 0.72 (0.49 - 1.06) | 0.298 |
| **Material status** |  |  |  |  |  |  |  |  |  |  |
| Single | 1 |  | 1 |  | 1 |  | 1 |  | 1 |  |
| Married | 0.97 (0.86 - 1.11) | 0.671 | 1.03 (0.89 - 1.19) | 0.681 | 1.01 (0.90 - 1.14) | 0.849 | 1.01 (0.87 - 1.17) | 0.914 | 0.95 (0.81 - 1.11) | 0.490 |
| Divorced/Widowed | 1.03 (0.86 - 1.23) | 0.762 | 1.07 (0.90 - 1.27) | 0.436 | 0.91 (0.77 - 1.07) | 0.262 | 1.07 (0.90 - 1.27) | 0.435 | 0.88 (0.70 - 1.11) | 0.290 |
| **Educational level** |  |  |  |  |  |  |  |  |  |  |
| Doctarate/Postdoctoral | 1 |  | 1 |  | 1 |  | 1 |  | 1 |  |
| Master’s degree | 0.92 (0.80 - 1.07) | 0.272 | 1.15 (0.96 - 1.37) | 0.130 | 1.01 (0.87 - 1.16) | 0.940 | 1.04 (0.87 - 1.24) | 0.702 | 1.09 (0.89 - 1.34) | 0.399 |
| Lato Sensu/MBA | 1.07 (0.93 - 1.24) | 0.347 | **1.37 (1.15 - 1.63)** | 0.001 | **1.19 (1.03 - 1.37)** | 0.018 | **1.28 (1.08 - 1.53)** | 0.005 | **1.27 (1.04 - 1.55)** | 0.018 |
| HS/HE/TPE* | 0.85 (0.65 - 1.10) | 0.213 | **1.31 (1.03 - 1.66)** | 0.028 | 1.10 (0.89 - 1.36) | 0.393 | 1.23 (0.95 - 1.60) | 0.122 | 1.17 (0.88 - 1.55) | 0.274 |
| **Region** |  |  |  |  |  |  |  |  |  |  |
| South | 1 |  | 1 |  | 1 |  | 1 |  | 1 |  |
| Southeast | 1.05 (0.88 - 1.25) | 0.611 | 1.09 (0.90 - 1.31) | 0.389 | 1.03 (0.88 - 1.21) | 0.721 | 1.06 (0.87 - 1.29) | 0.549 | 1.06 (0.86 - 1.30) | 0.613 |
| Midwest | 0.96 (0.80 - 1.15) | 0.629 | 0.96 (0.79 - 1.17) | 0.701 | 0.95 (0.80 - 1.12) | 0.517 | 1.00 (0.82 - 1.22) | 0.959 | 1.00 (0.81 - 1.24) | 0.980 |
| North and Northeast | 1.17 (0.98 - 1.38) | 0.077 | 1.15 (0.96 - 1.38) | 0.123 | 1.08 (0.92 - 1.25) | 0.356 | **1.23 (1.02 - 1.48)** | 0.028 | 1.16 (0.95 - 1.41) | 0.156 |
| **Position** |  |  |  |  |  |  |  |  |  |  |
| Teacher | 1 |  | 1 |  | 1 |  | 1 |  | 1 |  |
| Administrative-technical staff | 1.02 (0.91 - 1.14) | 0.756 | **1.23 (1.08 - 1.41)** | 0.002 | 1.10 (0.99 - 1.23) | 0.087 | **1.33 (1.15 - 1.53)** | <0.001 | **1.17 (1.01 - 1.36)** | 0.035 |
| **Lifestyle Habits** |  |  |  |  |  |  |  |  |  |  |
| Unhealthy dietary intake – considering the past 7 days | | | | | | | | | | |
| **UPF** |  |  |  |  |  |  |  |  |  |  |
| 0–4 days | 1 |  | 1 |  | 1 |  | 1 |  | 1 |  |
| 5–7 days | **1.27 (1.13 - 1.43)** | <0.001 | **1.19 (1.06 - 1.34)** | 0.003 | 1.07 (0.95 - 1.21) | 0.249 | **1.13 (1.00 - 1.27)** | 0.049 | **1.17 (1.03 - 1.34)** | 0.020 |
| **Soft drink** |  |  |  |  |  |  |  |  |  |  |
| 0–4 days | 1 |  | 1 |  | 1 |  | 1 |  | 1 |  |
| 5–7 days | 1.09 (0.91 - 1.30) | 0.349 | 1.02 (0.85 - 1.21) | 0.860 | 1.01 (0.86 - 1.18) | 0.904 | 0.91 (0.74 - 1.11) | 0.344 | 0.98 (0.80 - 1.20) | 0.843 |
| **Sweets** |  |  |  |  |  |  |  |  |  |  |
| 0–4 days | 1 |  | 1 |  | 1 |  | 1 |  | 1 |  |
| 5–7 days | 1.07 (0.96 - 1.19) | 0.252 | 1.04 (0.93 - 1.18) | 0.481 | 1.02 (0.93 - 1.12) | 0.699 | 0.98 (0.88 - 1.08) | 0.634 | 0.98 (0.86 - 1.10) | 0.707 |
| **Fried snacks** |  |  |  |  |  |  |  |  |  |  |
| 0–4 days | 1 |  | 1 |  | 1 |  | 1 |  | 1 |  |
| 5–7 days | 1.11 (0.82 - 1.51) | 0.491 | 0.99 (0.69 - 1.41) | 0.948 | 0.89 (0.64 - 1.22) | 0.461 | 1.03 (0.77 - 1.39) | 0.837 | 0.99 (0.67 - 1.46) | 0.952 |
| ***Fast food*** |  |  |  |  |  |  |  |  |  |  |
| 0–4 days | 1 |  | 1 |  | 1 |  | 1 |  | 1 |  |
| 5–7 days | **1.29 (1.01 - 1.65)** | 0.047 | 1.00 (0.73 - 1.37) | 0.992 | 0.87 (0.64 - 1.19) | 0.392 | 1.09 (0.84 - 1.42) | 0.528 | 1.25 (0.97 - 1.61) | 0.084 |
| Healthy dietary intake – considering the past 7 days | | | | | | | | | | |
| **Vegetables** |  |  |  |  |  |  |  |  |  |  |
| 5-7 days | 1 |  | 1 |  | 1 |  | 1 |  | 1 |  |
| 0-4 days | **1.20 (1.08 - 1.34)** | 0.001 | **1.23 (1.11 - 1.35)** | <0.001 | **1.14 (1.04 - 1.26)** | 0.005 | **1.16 (1.05 - 1.28)** | 0.005 | **1.23 (1.09 - 1.38)** | 0.001 |
| **Fruits** |  |  |  |  |  |  |  |  |  |  |
| 5-7 days | 1 |  | 1 |  | 1 |  | 1 |  | 1 |  |
| 0-4 days | **1.18 (1.06 - 1.31)** | 0.002 | **1.13 (1.03 - 1.24)** | 0.009 | 1.08 (0.99 - 1.18) | 0.094 | **1.13 (1.03 - 1.24)** | 0.014 | **1.13 (1.03 - 1.25)** | 0.012 |
| Sedentary behavior indicators |  |  |  |  |  |  |  |  |  |  |
| **Physical activity practice?** |  |  |  |  |  |  |  |  |  |  |
| Yes | 1 |  | 1 |  | 1 |  | 1 |  | 1 |  |
| No | **1.35 (1.21 - 1.49)** | <0.001 | **1.30 (1.18 - 1.43)** | <0.001 | **1.30 (1.18 - 1.42)** | <0.001 | **1.21 (1.10 - 1.33)** | <0.001 | **1.37 (1.23 - 1.52)** | <0.001 |
| **Physical activity weekly frequency** |  |  |  |  |  |  |  |  |  |  |
| ≥5 days | 1 |  | 1 |  | 1 |  | 1 |  | 1 |  |
| 3-4 days | 1.16 (0.96 - 1.40) | 0.157 | 1.04 (0.87 - 1.25) | 0.887 | 1.08 (0.92 - 1.28) | 0.354 | 1.07 (0.89 - 1.29) | 0.829 | 1.09 (0.88 - 1.34) | 0.699 |
| 1-2 days | 1.18 (0.95 - 1.47) | 0.149 | 1.12 (0.91 - 1.38) | 0.462 | 1.11 (0.91 - 1.36) | 0.253 | 1.15 (0.93 - 1.43) | 0.495 | 1.13 (0.90 - 1.43) | 0.399 |
| 0 days | **1.50 (1.27 - 1.78)** | <0.001 | **1.34 (1.16 - 1.57)** | <0.001 | **1.40 (1.21 - 1.63)** | <0.001 | **1.26 (1.07 - 1.49)** | 0.007 | **1.45 (1.21 - 1.74)** | <0.001 |
| **Television watching time** |  |  |  |  |  |  |  |  |  |  |
| ≤2 hours | 1 |  | 1 |  | 1 |  | 1 |  | 1 |  |
| 3–4 hours | 1.10 (0.95 - 1.27) | 0.210 | 1.12 (0.98 - 1.28) | 0.099 | 1.12 (0.99 - 1.26) | 0.085 | 0.97 (0.82 - 1.13) | 0.685 | 1.15 (0.99 - 1.33) | 0.069 |
| ≥5 hours | **1.28 (1.06 - 1.56)** | 0.010 | 1.13 (0.90 - 1.40) | 0.290 | **1.30 (1.09 - 1.56)** | 0.004 | 0.95 (0.72 - 1.24) | 0.660 | 1.04 (0.80 - 1.36) | 0.779 |
| **Computer use time** |  |  |  |  |  |  |  |  |  |  |
| ≤2 hours | 1 |  | 1 |  | 1 |  | 1 |  | 1 |  |
| 3–4 hours | 0.87 (0.65 - 1.17) | 0.365 | 1.05 (0.80 - 1.37) | 0.736 | 0.94 (0.75 - 1.17) | 0.557 | 0.97 (0.76 - 1.24) | 0.796 | 0.88 (0.67 - 1.16) | 0.328 |
| ≥5 hours | 1.05 (0.81 - 1.36) | 0.710 | 1.12 (0.88 - 1.43) | 0.373 | 1.02 (0.83 - 1.26) | 0.821 | 1.19 (0.94 - 1.50) | 0.142 | 1.14 (0.88 - 1.48) | 0.365 |
| Modifiable risk behaviors - Considering the past 30 days | | | | | | | | | | |
| **Cigarette consumption** |  |  |  |  |  |  |  |  |  |  |
| 0 days | 1 |  | 1 |  | 1 |  | 1 |  | 1 |  |
| 1–9 days | 0.77 (0.48 - 1.24) | 0.279 | 1.09 (0.84 - 1.41) | 0.518 | 0.85 (0.59 - 1.22) | 0.386 | 0.91 (0.62 - 1.33) | 0.614 | 0.91 (0.61 - 1.34) | 0.618 |
| ≥10 days | 0.96 (0.70 - 1.31) | 0.773 | 0.88 (0.65 - 1.18) | 0.394 | 0.85 (0.61 - 1.19) | 0.347 | 0.97 (0.69 - 1.35) | 0.838 | 0.90 (0.63 - 1.27) | 0.533 |
| **Alcohol consumption** |  |  |  |  |  |  |  |  |  |  |
| 0 days | 1 |  | 1 |  | 1 |  | 1 |  | 1 |  |
| 1–2 days | 0.99 (0.86 - 1.13) | 0.837 | 0.90 (0.79 - 1.03) | 0.131 | 0.93 (0.83 - 1.05) | 0.262 | 0.89 (0.79 - 1.02) | 0.085 | 0.97 (0.84 - 1.12) | 0.681 |
| 3–9 days | 0.93 (0.81 - 1.06) | 0.282 | 0.97 (0.85 - 1.11) | 0.654 | **0.88 (0.78 - 0.99)** | 0.039 | 0.96 (0.85 - 1.08) | 0.485 | 0.92 (0.79 - 1.06) | 0.253 |
| ≥10 days | 0.91 (0.76 - 1.09) | 0.321 | 0.95 (0.80 - 1.13) | 0.562 | **0.81 (0.98 - 0.96)** | 0.018 | **0.81 (0.67 - 0.98)** | 0.027 | **0.79 (0.64 - 0.97)** | 0.027 |
| **Number of drinks per drinking day** |  |  |  |  |  |  |  |  |  |  |
| 0 drinks | 1 |  | 1 |  | 1 |  | 1 |  | 1 |  |
| <1 drink | 0.90 (0.71 - 1.14) | 0.386 | 0.95 (0.78 - 1.17) | 0.627 | 0.91 (0.73 - 1.12) | 0.368 | 0.94 (0.78 - 1.14) | 0.517 | 1.03 (0.85 - 1.25) | 0.772 |
| 1–2 drinks | 0.96 (0.84 - 1.09) | 0.511 | 0.89 (0.78 - 1.02) | 0.102 | 0.94 (0.84 - 1.06) | 0.317 | **0.84 (0.74 - 0.96)** | 0.010 | 0.93 (0.81 - 1.08) | 0.359 |
| 3–4 drinks | 0.90 (0.77 - 1.06) | 0.201 | 0.95 (0.82 - 1.10) | 0.480 | **0.84 (0.73 - 0.97)** | 0.019 | 0.92 (0.80 - 1.07) | 0.281 | **0.82 (0.69 - 0.98)** | 0.032 |
| ≥5 drinks | 1.01 (0.87 - 1.18) | 0.916 | 1.02 (0.88 - 1.20) | 0.771 | 0.89 (0.76 - 1.04) | 0.133 | 0.94 (0.80 - 1.11) | 0.475 | 0.97 (0.81 - 1.15) | 0.720 |
| **Mental health conditions** |  |  |  |  |  |  |  |  |  |  |
| **Depression** |  |  |  |  |  |  |  |  |  |  |
| Normal | 1 |  | 1 |  | 1 |  | 1 |  | 1 |  |
| Mild/Moderate | **1.88 (1.66 - 2.13)** | <0.001 | **1.94 (1.71 - 2.20)** | <0.001 | **1.47 (1.31 - 1.64)** | <0.001 | **1.52 (1.36 - 1.70)** | <0.001 | **2.05 (1.77 - 2.37)** | <0.001 |
| Severe/Extremely severe | **2.30 (2.04 - 2.59)** | <0.001 | **2.31 (2.03 - 2.64)** | <0.001 | **1.75 (1.57 - 1.95)** | <0.001 | **1.90 (1.69 - 2.14)** | <0.001 | **2.60 (2.24 - 3.01)** | <0.001 |
| **Anxiety** |  |  |  |  |  |  |  |  |  |  |
| Normal | 1 |  | 1 |  | 1 |  | 1 |  | 1 |  |
| Mild/Moderate | **1.53 (1.34 - 1.74)** | <0.001 | **1.42 (1.25 - 1.61)** | <0.001 | **1.33 (1.19 - 1.49)** | <0.001 | **1.38 (1.22 - 1.55)** | <0.001 | **1.51 (1.31 - 1.74)** | <0.001 |
| Severe/Extremely severe | **1.91 (1.70 - 2.14)** | <0.001 | **1.75 (1.55 - 1.97)** | <0.001 | **1.65 (1.50 - 1.82)** | <0.001 | **1.69 (1.50 - 1.91)** | <0.001 | **1.97 (1.72 - 2.25)** | <0.001 |
| **Stress** |  |  |  |  |  |  |  |  |  |  |
| Normal | 1 |  | 1 |  | 1 |  | 1 |  | 1 |  |
| Mild/Moderate | **1.66 (1.46 - 1.89)** | <0.001 | **1.60 (1.42 - 1.80)** | <0.001 | **1.42 (1.27 - 1.58)** | <0.001 | **1.37 (1.22 - 1.53)** | <0.001 | **1.67 (1.45 - 1.92)** | <0.001 |
| Severe/Extremely severe | **2.06 (1.83 - 2.31)** | <0.001 | **1.95 (1.72 - 2.20)** | <0.001 | **1.65 (1.49 - 1.83)** | <0.001 | **1.74 (1.54 - 1.95)** | <0.001 | **2.27 (1.99 - 2.59)** | <0.001 |

**Note:** The table presents the results of the bivariate analysis between the independent variables and the outcome of low QoL, defined based on the median of each WHOQOL-bref domain. Scores below the median were considered as low QoL. Adjusted Relative Risk (RRadj) estimates were obtained using Generalized Estimating Equations (GEE) models. * “HS” is an abbreviation for high school, “HE” for higher education, “TPE” for technical professional education, and “MBA” for Master of Business Administration.
